# *Cryptococcus gattii* in AIDS Patients, Southern California

**DOI:** 10.3201/eid1111.040875

**Published:** 2005-11

**Authors:** Sudha Chaturvedi, Madhu Dyavaiah, Robert A. Larsen, Vishnu Chaturvedi

**Affiliations:** * Wadsworth Center, Albany, New York, USA; † State University of New York, Albany, Albany, New York, USA; ‡ University of Southern California, Los Angeles, California, USA; § Los Angeles County Hospital, Los Angeles, California, USA

**Keywords:** *Cryptococcus gattii*, *Cryptococcus neoformans* varieties *grubii* and *neoformans*, pheromones, CE-FLA, CE-SSCP, southern California, AIDS, molecular methods, research

## Abstract

*Cryptococcus* isolates from AIDS patients in southern California were characterized by molecular analyses. Pheromone *MFα1* and *MF****a***1 gene fragments were polymerase chain reaction–amplified with fluorescently labeled primers and analyzed by capillary electrophoresis (CE) on DNA analyzer. CE–fragment-length analyses (CE-FLAs) and CE–single-strand conformation polymorphisms (CE-SSCPs) were used to determine *Cryptococcus gattii* (*Cg*), *C. neoformans* (*Cn*) varieties *neoformans* (*CnVN*) and *grubii* (*CnVG*), mating types, and hybrids. Corroborative tests carried out in parallel included growth on specialized media and serotyping with a commercial kit. All 276 clinical strains tested as haploid *MATα* by CE-FLA. CE-SSCP analyses of *MFα1* showed 219 (79.3%) *CnVG*, 23 (8.3%) *CnVN*, and 34 (12.3%) *Cg* isolates. CE-FLA and CE-SSCP are promising tools for high-throughput screening of *Cryptococcus* isolates. The high prevalence of *Cg* was noteworthy, in view of its sporadic reports from AIDS patients in North America and its recent emergence as a primary pathogen on Vancouver Island, Canada.

## Abstract 2

A molecular analysis of pheromone genes showed a notable prevalence of *Cryptococcus gattii* isolates from AIDS patients in southern California.

The encapsulated basidiomycete *Cryptococcus neoformans* was recently divided into 2 species, *C. neoformans* (*Cn*) and *C*. *gattii* (*Cg*) (*1*). *Cn* consists of 2 varieties, *grubii* (*CnVG*) and *neoformans* (*CnVN*), which are opportunistic pathogens and predominantly infect immunocompromised persons (*2**,**3*). *CnVG* is the major causative agent of cryptococcosis worldwide, except in central Europe, where *CnVN* infection is most prominent. In contrast, *Cg* is a primary pathogen, which predominantly infects immunocompetent persons (*4*). *Cg* was previously thought to be restricted to tropical and subtropical climates with a special ecologic niche on *Eucalyptus* trees (*5**,**6*). However, the recent outbreak of *Cg* infection in healthy humans and animals in the temperate climate of Vancouver Island, British Columbia, Canada, and its isolation from several species of trees other than *Eucalyptus* have raised the strong possibility that this fungus might have broader geographic distribution (*7**–**9*).

The mechanisms underlying pathogenic and environmental differences between *Cn* and *Cg* are not known. Within *Cn* species, *CnVN* infections are more likely to display skin involvement and to afflict older patients, whereas *CnVG* infections are reported to cause a higher mortality rate (*3**,**10*). In contrast, infections caused by *Cg* result in a lower mortality rate but are frequently complicated by neurologic sequelae and require surgery and prolonged therapy (*4**,**11*). Our recent studies with the Cu,Zn SOD (*SOD1*) and MnSOD (*SOD2*) knockout mutants of *Cg* indicated that these antioxidants are crucial for *Cg* pathogenesis (*12**,**13*). In contrast, the antioxidant function of *SOD1* in *CnVG* is less crucial for pathogenesis (*14*). These observations are the first molecular evidence of a likely divergence in the pathogenic mechanisms used by *Cn* and *Cg*.

Both *Cn* and *Cg* have a single locus, 2-allele mating system comprising *MATα* and *MAT****a*** strains. The *MATα* strains of *Cn* and *Cg* predominate in nature and in clinical settings, and this predominance over *MAT****a*** strains is linked to high virulence and reproduction by haploid fruiting (*3**,**15*). Generally, *Cryptococcus* strains are haploid, but hybrid strains have also been characterized from both clinical and environmental sources (*16**–**20*). Thus, characterizing clinical *Cryptococcus* isolates to the individual species or varieties and according to mating and hybrid types could be useful for managing cryptococcosis cases and for further understanding the epidemiology of this disease.

Several laboratory typing methods have been used in epidemiologic studies of cryptococcosis, including serotyping, electrophoretic karyotyping, use of mitochondrial DNA probes, use of genomic DNA probes, determination of allelic variations at the *URA5* locus, multilocus enzyme typing, measurement of creatinine utilization, polymerase chain reaction (PCR), fingerprinting and amplified fragment-length polymorphism (reviewed in [2]). Previously, we described a PCR–restriction fragment length polymorphism (PCR-RFLP) typing scheme for *Cn* and *Cg* pheromone genes, which could be used for characterizing mating types, hybrids, and variety (*21*). In the present study, we developed a capillary electrophoresis–fragment length analysis (CE-FLA) test, and a CE–single stranded conformation polymorphism (CE-SSCP) test by using the pheromone genes *MFα1* and *MF****a****1*. These tests were used in parallel with more traditional specialized culture medium and a commercial serotyping kit to characterize *Cryptococcus* isolates from AIDS patients in southern California.

## Materials and Methods

### *Cryptococcus* Isolates

Two hundred seventy-six *Cryptococcus* isolates originating from patients with HIV/AIDS were obtained from the Infectious Diseases Laboratory, Los Angeles County Hospital, Los Angeles, California. The isolates were suspended in sterile skim milk and stored at –20°C. The isolates were transferred frozen to the Mycology Laboratory of the Wadsworth Center in Albany, New York, USA, where they were streaked on Niger seed agar plates (*3*) to check for purity and reconfirmation of their identity; a typical colony was picked for further analysis. The subcultures were placed in long-term storage in sterile 15% glycerol at –70°C. These isolates were further characterized in our laboratory by testing their growth on canavanine-glycine-bromothymol blue (CGB) agar for differentiation of *Cryptococcus* species (*22*) and serotyping with Crypto Check Kit (Iatron Laboratories Inc., Tokyo, Japan). Several investigators gave strains to put together a panel of reference isolates that were either currently being used in molecular pathogenesis studies, represented type strains, or were otherwise unique. The details of these 16 reference isolates are listed in Table 1. Six additional A/D hybrid strains, characterized in our earlier study, were also used (*18*).

**Table 1:** Cryptococcus neoformans (Cn) and Cryptococcus gattii (Cg) strains used in this study for standardization of reagents*

Strain identity	Variety/species	Mating type	Source
H99 (NYSD 1649)	*CnVG*	α	New York State Herbarium, Albany, NY
KN99α	*CnVG*	α	J. Heitman, Duke University, Durham, NC
KN99**a**	*CnVG*	**a**	J. Heitman, Duke University, Durham, NC
IUM96–2828	*CnVG*	**a**	B.L. Wickes, University of Texas Health Sciences Center, San Antonio, TX
NIH12 (ATCC 28959)	*CnVN*	α	ATCC, Manassas, VA
JEC21	*CnVN*	α	J.C. Edman, University of California San Francisco (UCSF), San Francisco, CA
JEC20	*CnVN*	**a**	J.C. Edman, UCSF, San Francisco, CA
NIH430 (ATCC 28958)	*CnVN*	**a**	ATCC, Manassas, VA
NIH433 (ATCC 34875)	*CnVN*	**a**	ATCC, Manassas, VA
NIH444 (ATCC 32609)	*Cg*	α	ATCC, Manassas, VA
NIH191 (ATCC 32608)	*Cg*	**a**	ATCC, Manassas, VA
NIH198	*Cg*	**a**	K.J. Kwon-Chung, National Institutes of Health, Bethesda, MD
WM0135	*Cg*	**a**	W. Meyer, University of Sydney, Sydney, Australia
WM-138	*Cg*	**a**	W. Meyer, University of Sydney, Sydney, Australia
UM2	Hybrid (A/D)	α/**a**	F. Dromer, Institute Pasteur, Paris, France
UM8	Hybrid (A/D)	α/**a**	F. Dromer, Instotite Pasteur, Paris, France

*ATCC, American Type Culture Collection; *VG*, var. *grubiii*; *VN*, var. *neoformans*.

### Multiplex PCR for Pheromone Genes

**Figure 1:**
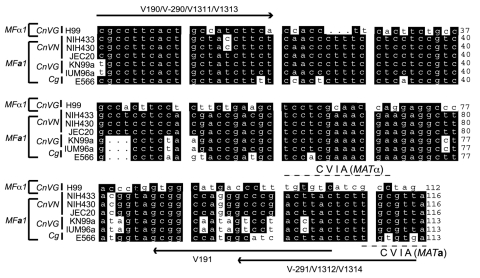
Primers for pheromone polymerase chain reaction (PCR). Nucleotide sequence alignment for MFα1 and MFa1 genes is shown with characteristic Cys-Val-Ile-Ala (CVIA) motifs. Both sense and antisense primers were designed from within the open reading frames of pheromone genes, to ensure high specificity of the multiplex PCR. The MFα1 sequence from Cryptococcus neoformans var. grubii (CnVG) (AF542529) and the MFa1 sequences from CnVG (AY129299), Cryptococcus neoformans var. neoformans (CnVN) (AF542530), and Cryptococcus gattii (Cg) (AY710429) were used for multiple alignments with GCG (Wisconsin package version 10.0). A common primer pair, V190/V191, was designed to get MFα1 PCR amplicons from CnVG, CnVN, and Cg (MFα1 sequence from CnVG was used as a reference), while unique primer pairs V290/V291, V1311/V1312, and V1313/V1314 were designed to get MFa1 PCR amplicons from CnVN, CnVG, and Cg, respectively. All the 3´-PCR primers contained a sequence from CVIA motif, which provided specificity to PCRs for pheromone genes.

A previous report from this laboratory described the use of specific primers for amplification of *MFα1* and *MF****a****1* gene fragments, which could be separated as 100-bp and 117-bp fragments on a specialized agarose gel (*21*). The primer sets V290/V291, which was earlier designed to amplify *MF****a****1* gene from *CnVN*, did not amplify similar genes from *MAT****a*** strains of either *CnVG* or *Cg*. Multiple alignment of *MF****a****1* indicated that this gene is highly polymorphic among *CnVG*, *CnVN*, and *Cg* (Figure 1). Therefore, 2 new sets of primers were designed to obtain *MF****a****1* amplicons from *CnVG* and *Cg*. These primers are listed in Table 2. A multiplex PCR for simultaneous amplification of *MFα1* and *MF****a****1* in a 50-μL reaction volume was performed with 5 μL of 10× PCR buffer with 15 mmol/L MgCl_2_, 2.5 μL of each of 8 primers (10 μmol/L stock), 3.0 μL dNTP mix (10 μmol/L each), and 2.0 U Taq DNA polymerase (Perkin Elmer, Foster City, CA, USA). The template DNA was 5.0 μL of either a boiled cell suspension or 50 ng genomic DNA. Initial denaturation was conducted at 95°C for 3 min, followed by 30 cycles of denaturation at 94°C for 1 min, annealing at 57.5°C for 1 min, amplification at 72°C for 1 min, and final extension at 72°C for 7 min, in a GeneAmp PCR System 9600 (Perkin Elmer). In preliminary experiments, PCR products (10-μL aliquots) were resolved by electrophoresis on 3.5% MetaPhor agarose (FMC Bio-Products, Rockland, ME, USA) gels in Tris-borate-EDTA (TBE) buffer, and were detected by ethidium bromide staining. The PCR experiments were repeated twice, and identical results were obtained.

**Table 2:** Primers used in this study*

Primer name	Sequence	Target	Source/reference
V190–5´	5´-CTTCACTGCCATCTTCACCA-3´	*MFα1*–*Cg*, *CnVN*, and *CnVG*	(21)
V191–3	5´-GACACAAAGGGTCATGCCA-3´		
V290–5´	5´-CGCCTTCACTGCTACCTTCT-3´	*MF****a****1*–*CnVN*	(21)
V291–3´	5´-AACGCAAGAGTAAGTCGGGC-3´		
V1311–5´	5´-TGCCTTCACTGCTATCTTCT-3´	*MF****a****1*–*CnVG*	This study
V1312–3´	5´-AACGCAAGAGTAGGTAGGAC-3´		
V1313–5´	5´-CGCCTTCACTGCTATCTTTTC-3´	*MF****a****1*–*Cg*	This study
V1314–3´	5´-CACACAAGAGTAAGTGATGC-3´		

*Cn, *Cryptococcus neoformans*; *Cg*, *Cryptococcus gattii*; *VN*, var. *neoformans*; *VG*, var. *grubii*.

### Gene Scan Analysis

The *MFα1* sense primer (V190) was labeled with FAM (6-carboxyfluorescein) at the 5´ end, the antisense primer (V191) was labeled with tetrachloro-fluorescein (TET) at the 3´ end, and *MF****a****1* sense primers (V290, V1311, V1313) were labeled with 6-carboxy-2´, 4´, 4´, 5´, 7´, 7´-hexachlorofluorescein (HEX) at the 5´ end. The fluorescent dye–labeled primers were custom ordered (Operon Technologies, Inc., Alameda, CA, USA). FLA and SSCP of the *MFα1* and *MF****a****1* PCR amplicons were determined by CE with an ABI PRISM 310 Genetic Analyzer, and the electronic images were analyzed by using GeneScan analysis software (Applied Biosystems Inc., Foster City, CA, USA). The sample preparation for CE consisted of 1 μL *MFα1* and *MF****a****1* PCR amplicons, 12 μL highly deionized formamide, and 0.5 μL GeneScan-500 (TAMRA) size standard (Applied Biosystems). The sample mixture was denatured for 5 min at 95°C and was then rapidly cooled on ice before loading on the instrument. For CE-FLA, the samples were analyzed under denaturing conditions (POP-4 polymer [Applied Biosystems] in buffer supplied by manufacturer) at 60°C, and for CE-SSCP, the samples were analyzed under nondenaturing conditions (3% GeneScan polymer in 1× TBE buffer with 10% glycerol) at 30°C. A capillary (47 cm × 50 μm inside diameter) was installed, and POP-4 or 3% polymer was filled according to manufacturer's instructions. The electrophoresis conditions for CE-FLA were 5-s injection time, 15-kV injection voltage, 15-kV electrophoresis voltage, 150-s syringe pump time, 120-s preinjection electrophoresis, and 20-min collection time for each sample, and the run was performed at 60°C. The electrophoresis conditions for CE-SSCP were 5-s injection time, 15-kV injection voltage, 13-kV electrophoresis voltage, 30-s syringe pump time with no preinjection time, and 20-min collection time for each sample, and the run was performed at 30°C. CE-FLA and CE-SSCP standardization experiments were carried out on >4 independent occasions, and unknown sample analyses were repeated at least once.

## Results

### Multiplex PCR

**Figure 2:**
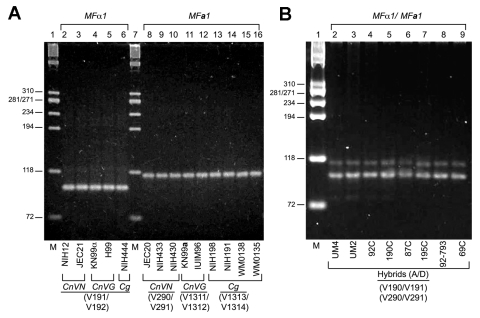
Multiplex polymerase chain reaction (PCR) for pheromone fragment analysis. A) Multiplex PCR with 4 sets of primers comprising MFα1 (V190/V191) and MFa1 (V290/V291, V1311/V1312, V1313/V1314) genes were carried out as described in Materials and Methods. Approximately 100-bp MFα1 and 117-bp MFa1 PCR amlicons were detected on 3.5% MetaPhor agarose in Tris-borate-EDTA buffer for MATα and MATa strains comprising Cryptococcus neoformans var. grubii (CnVG), Cryptococcus neoformans var. neoformans (CnVN), and Cryptococcus gattii (Cg). Lanes 1 and 7, molecular mass marker. B) Multiplex PCR depicting MFα1 and MFa1 PCR amplicons from the 8 known hybrid (A/D) isolates. Lane 1, molecular mass marker.

The 4 sets of primers (*MFα1*/*MF****a****1*) produced reproducible results for control *CnVG*, *CnVN*, *Cg* haploids (Figure 2A), and A/D hybrid strains (Figure 2B). These results validated the robustness of the primers, which had been designed from well within the open reading frames of 2 pheromone genes, to prevent amplification of any nontarget DNA. The latter objective also informed the decision to anchor the 3´ ends of all PCR primers within the characteristic Cys-Val-Ile-Ala (CVIA) motifs; this eliminated any possible amplification of other pheromone genes since this is the only sequence shared among fungal pheromones (*18*). Even though multiple copies of *MFα* and *MF****a*** genes have been reported in *C*. *gattii* and *C. neoformans* by Southern hybridization and whole genome-sequencing, PCR primers only amplify single amplicons because these genes have identical nucleotide sequences (*18**,**23**,**24*). Although this multiplex method was well suited for identifying mating types and hybrids, further delineation of species and varieties would require restriction digestion with several unique enzymes as we stated previously (*21*). Therefore, we decided to use CE-FLA and CE-SSCP to further characterize pheromone gene amplicons. These techniques have been successfully used to delineate fragment length as well gene mutations for characterizing various fungal and bacterial isolates (*25**–**27*). The SSCP analysis displays migration of the amplified DNA fragment as a function of that fragment's structural conformation. Given that the tertiary structure of a fragment is sensitive to single nucleotide substitutions, this method was shown to be suitable for detecting single nucleotide changes when 100-bp to 300-bp DNA fragments were analyzed (*28*). Since amplified pheromone fragments yield ≈100- to 120-bp products, they were an ideal substrate for this method of mutant detection.

### CE-FLA

**Figure 3:**
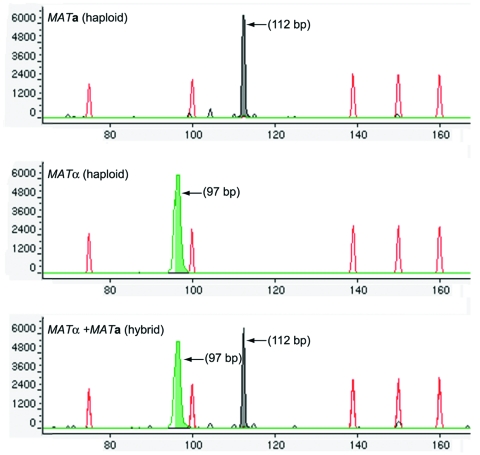
Capillary electrophoresis fragment-length analyses (CE-FLA) for the identification of mating types and hybrids. The ABI PRISM 310 Genetic Analyzer and GeneScan analysis software were used for the fragment length analysis of the pheromone genes. Sense strands of MFα1 and MFa1 were labeled with fluorescent probes TET (green) and HEX (black), respectively, and polymerase chain reaction amplicons were analyzed with POP-4 polymer under denaturing conditions at 60°C. Green peak, MFα1; black peak, MFa1. These peaks were aligned by using an internal size standard, GeneScan-500 TAMRA (red peaks).

The 16 reference strains of known *Cryptococcus* species, varieties, mating types, and hybrids were used to establish a robust CE-FLA protocol with denaturing POP-4 polymer at 60°C. The electrophoretic runs with POP-4 polymer produced a 112-bp DNA fragment for *MF****a****1* and 97-bp fragment for *MFα1*, which were easily distinguished with the GeneScan software by the characteristic peak sizes (Figure 3). CE-FLA allowed *Cryptococcus* mating types and hybrids to be identified, but not *CnVG*, *CnVN*, and *Cg*.

### CE-SSCP

**Figure 4:**
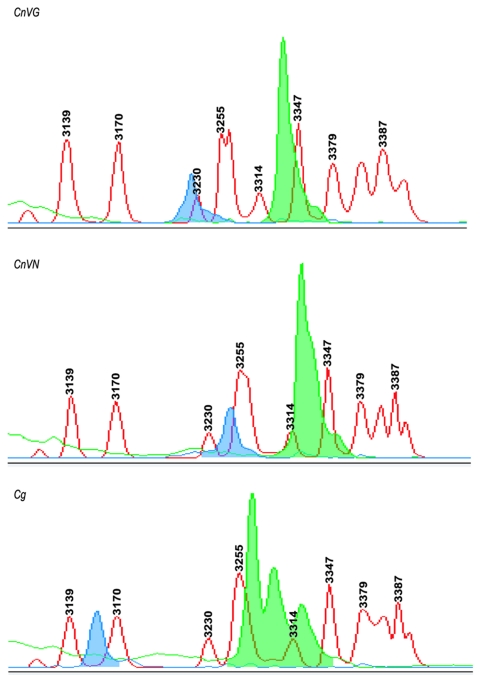
Capillary electrophoresis–single strand conformation polymorphisms (CE-SSCP) for the identification of varieties and species. The ABI PRISM 310 Genetic Analyzer and GeneScan analysis software were used for variety and species determination with the MFα1 pheromone gene. The MFα1 sense and antisense primers were labeled with fluorescent probes FAM (blue) and TET (green), and polymerase chain reaction amplicons were analyzed with 3% polymer at 30°C under nondenaturing conditions. The blue and green peaks depict characteristic peak pattern for Cryptococcus neoformans var. grubii (CnVG), Cryptococcus neoformans var. neoformans (CnVN), and Cryptococcus gattii (Cg). These peaks were aligned by using an internal size standard.

CE-SSCP test under nondenaturing conditions with 3% GeneScan polymer at 30°C allowed characteristic peak patterns to be detected in *MFα1* and *MF****a****1* genes because of the individual differences within the nucleotide sequences. Distinct patterns obtained for *CnVG*, *CnVN*, and *Cg* by using *MFα1* gene are shown in Figure 4. The sense strand (labeled blue) yielded 1 characteristic peak pattern, while the antisense strand (labeled green) yielded 2–3 characteristic peak patterns. We subsequently decided to label only the sense strand to reduce the cost of the PCR primers as well the complexity of the peak patterns observed with antisense strand. For determining an unknown sample, the instrument analyses needed to yield highly reproducible results. Therefore, a sample of each of the *Cryptococcus* strains was injected on 4 separate occasions into the same capillary, and the precision of the sizing was calculated. The low standard deviations associated with each mean peak value indicated that the assignment of variety or species for an unknown sample, based on pattern sizing information alone, would be highly reliable (Table 3).

**Table 3:** Calibration of SSCP peak positions for CnVG, CnVN, and Cg*

*Cn* strains	Sense strand peak†
*CnVG* (KN99α; *MATα*)	3230.57 ± 0.37
*CnVG* (KN99**a**; *MAT****a***)	4501.35 ± 1.29
*CnVN* (NIH12; *MATα*)	3252.77 ± 1.29
*CnVN* (NIH430; *MAT****a***)	4643.75 ± 1.12
*Cg* (NIH 444; *MATα*)	3161.54 ± 0.95
*Cg* (NIH198; *MAT****a***)	4593.35 ± 1.2

*SSCP, single-strand conformation polymorphisms; *Cn*, *Cryptococcus neoformans*; *Cg*, *Cryptococcus gattii*; *VN*, var. *neoformans*; *VG*, var. *grubii*. †Mean ± SD of 4 independent experiments.

Based on our success with *MFα1* sense primer in the detection of characteristic peaks for *CnVG*, *CnVN*, and *Cg*, we labeled *MF****a****1* sense strands and analyzed *MAT****a*** strains. In this case, we had to use individual sets of *MF****a****1* primers because of the substantial polymorphism observed at the 5´ and 3´ end of this gene between *Cg* and *Cn*, and within *Cn* vatietie*s* (Figure 1). Again, the *MF****a****1* peak pattern was unique to each *Cn* variety and *Cg* (Table 3). Overall, our results indicated that either *MFα1* or *MF****a****1* gene products yielded unique SSCP patterns and could be used for identifying *Cn* varieties and *Cg* strains.

### California Isolates

We used standardized CE-FLA and CE-SSCP techniques to analyze 276 isolates of *Cryptococcus* that were obtained from AIDS patients and that were stored at the Infectious Diseases Laboratory, Los Angeles County Hospital, Los Angeles, California. The investigations were fully compliant with Los Angeles County Hospital–University of Southern California (USC) Institutional Review Board guidelines (proposal #924008). CE-FLA showed that all 276 isolates were *MATα* strains, and no *MAT****a*** or hybrid strains were found in our samples. CE-SSCP found that among the total 276 clinical isolates, 219 (79.3%) were *CnVG*, 23 (8.3%) were *CnVN*, and 34 (12.3%) were *Cg*. For corroborations, all of these isolates were also tested by growth on CGB agar, and by serotyping with the Crypto Check Kit, both of which yielded results in agreement with those obtained with pheromone typing.

## Discussion

The relatively high prevalence of *Cg* in this survey is noteworthy for several reasons (Table 4). First, we believe it is the first instance in which a large number of *Cg* clinical isolates from AIDS patients have been identified in the United States. Second, *Cg* has never been considered a substantial cause of cryptococcosis among US AIDS patients, including those in the southern California. Third, the presence of *Cg* in HIV-AIDS patient samples in the USC collection is similar to the prevalence recently reported from some countries in Central and South America (*29*), and it contrasts with the rare occurrence of *Cg* in immunocompromised patient populations in Australia, Southeast Asia, and Africa (*30**–**32*). The prevalence of *CnVN* (8%) in our samples closely matches its recently reported prevalence in New York City (*33*). Thus, cryptococcosis due to *CnVN* in AIDS patients is not a rare clinical entity in the United States, and its pattern of distribution on the East and West Coasts does not differ. The absence of *MAT****a*** strains in our samples is not surprising, in view of the rare occurrence of this mating type among clinical and environmental specimens (*34*). This finding is consistent with the results of other recent clinical and environmental surveys in the United States, Europe, and Australia. The *Cg* outbreak on Vancouver Island also yielded only *MATα* isolates (*8**,**35*). Overall, our inferences are based on limited data, since most published studies on cryptococcosis do not include detailed characterization of *Cryptococcus* strains. Future epidemiologic studies will likely yield a more complete picture of the causative varieties or species of *Cryptococcus* across the United States.

**Table 4:** Relative distribution of CnVG, CnVN, and Cg in HIV-AIDS patients from southern California

Isolates*	n (%) (N = 276)
*CnVG*	219 (79.3)
*CnVN*	23 (8.3)
*Cg*	34 (12.3)

*Cn, *Cryptococcus neoformans*; *Cg*, *Cryptococcus gattii*; *VN*, var. *neoformans*; *VG*, var. *grubii*.

As previously noted, *CnVG* infections are predominant in AIDS patients around the world, except in Europe, where *CnVN* is seen in sizable numbers. One explanation for this phenomenon is that *CnVG* is best adapted for the colonization of soil and pigeon droppings. However, *CnVN* may dominate the same ecologic niche in parts of Europe for undetermined causes (*10*). The lower incidence of *Cg* infection in AIDS patients could be due to the paucity of regions around the world in which *Cg* is endemic and the reported association of *Cg* disease with the flowering of *Eucalyptus camaldulensis* trees in certain areas (*6*). The unprecedented outbreak of *Cg* infection in Vancouver Island already comprises 66 human and 50 animal cases of cryptococcosis. Ongoing investigation in Vancouver Island indicate that the numbers of human and animal cases are increasing (130 human cases and >200 animal cases), which led to the recent change in the definition of *Cg* outbreak to *Cg* endemicity in this region (*7*). Additionally, *Cg* was isolated from swab samples from the bark of trees of many species (alder, arbutus, bitter cherry, cedar, fir, garry oak, maple, spruce), as well as from soil and air samples near these trees (*7*). These investigations have added a new dimension to our understanding of *Cg* ecology and suggest that this pathogen is neither restricted in its geographic distribution nor to its presumed natural host, *Eucalyptus* trees.

Our results indicated that both CE-FLA and CE-SSCP of pheromone genes are amenable to semi-automation and large-scale analyses of pathogenic *Cryptococcus* species, varieties, mating types, and hybrids. Each step of this analysis, namely, PCR, heat denaturation with formamide, and subsequent loading of samples, can be carried out in 48- or 96-well trays, which allow the use of multichannel or automated pipettors. Both CE-FLA and CE-SSCP individual runs are completed in <20 min, and the instrument can be programmed for multiple runs, thereby giving a high throughput. Thus, analyzing hundreds to thousands of strains is a good possibility, especially in reference laboratories. Moreover, the electrophoretic runs are saved as electronic files for easy portability over the Internet and to facilitate interlaboratory comparisons. This study reports a logical improvement over our earlier published method on pheromone PCR-RFLP for characterizing *Cryptococcus* isolates. The use of CE-FLA and CE-SSCP allowed us to dispense with running specialized gels as well as the use of unique restriction digestion schemes (*21*). Thus, CE-FLA alone leads to visualization of size differences in *MFα1* versus *MF****a****1* pheromones, which would distinguish mating types and hybrids. The species and varieties could be distinguished by CE-SSCP on the basis of polymorphisms in nucleotide sequences of *MFα1* and *MF****a****1* in *Cg*, *CnVG*, and *CnVN*. Thus, 1 typing method had the potential to replace multiple tests, such as specialized media and serotyping kits for species/variety determination, crossing with tester strains on mating agar, and flow cytometry for hybrid determination. The current limitations of this approach include the use of 2 polymers and run temperatures, which makes it necessary to run CE-FLA and CE-SSCP as batch applications on ABI 310 Genetic Analyzer. Since individual electrophoretic runs are completed in ≈20 min, ≈16 hours will be necessary to analyze ≈45 samples (one 48-sample tray) by CE-FLA, followed by change of polymer and run conditions, and another 16 hours to complete CE-SSCP analyses. However, these limitations could be easily overcome in the upgraded model of this instrument (ABI 3130), which has 4–16 capillaries and hands-free, 24-hour operation capabilities for simultaneous analyses of multiple samples, thereby considerably decreasing turnaround time.
